# Diffuse hypertrophic obstructive cardiomyopathy complicated by apical ventricular aneurysm and excessive left ventricular trabeculation: a case report

**DOI:** 10.3389/fcvm.2026.1753832

**Published:** 2026-04-22

**Authors:** Ying Li, Na Jia, Xinyue Li, Jingwen Dai, Deping Liu

**Affiliations:** 1 Peking University Fifth School of Clinical Medicine, Beijing, China; 2 Department of Cardiology, Beijing Hospital, National Center of Gerontology, Institute of Geriatric Medicine, Chinese Academy of Medical Sciences, Beijing, China; 3 Department of General Practice, Taihu Community Health Service Center, Beijing, China; 4 Department of Radiology, Beijing Hospital, National Center of Gerontology, Institute of Geriatric Medicine, Chinese Academy of Medical Sciences, Beijing, China

**Keywords:** hypertrophic cardiomyopathy, left ventricular apical aneurysm, left ventricular excessive trabeculation, MYBPC3, ventricular arrhythmia

## Abstract

This article reports a rare case of diffuse hypertrophic obstructive cardiomyopathy complicated with left ventricular apical aneurysm and excessive trabeculation. Genetic testing of the patient revealed a heterozygous variant of the MYBPC3 gene (c.3343G> A: p.Val1115Ile). Despite optimal medical therapy and implantable cardioverter-defibrillator (ICD) implantation, the patient developed progressive cardiac dysfunction and recurrent ICD shocks. We discuss the clinical course, genetic findings, and imaging features of this case.

## 1 Introduction

Hypertrophic cardiomyopathy (HCM) is a common genetic heart disease with heterogeneous phenotypes. Unlike classic basal asymmetrical septal HCM, approximately 42%–54% of HCM cases exhibit diffuse symmetric myocardial hypertrophy, characterized by concentric myocardial hypertrophy without regional preferences (1–3). Left ventricular apical aneurysm (LVAA) is a rare but severe complication of HCM. It occurs in approximately 2%–5% of HCM patients and is associated with an increased risk of sudden cardiac death (SCD), thromboembolism, and heart failure exacerbation (4). Additionally, the coexistence of left ventricular excessive trabeculation (LVET) further complicates the clinical course (5). Notably, the coexistence of diffuse hypertrophic obstructive cardiomyopathy, LVAA, and LVET is exceptionally uncommon and has been rarely reported. Moreover, our case was further characterized by a unique MYBPC3 missense variant and long-term progressive deterioration to end-stage heart failure. These features make the present case highly unique and clinically noteworthy, providing valuable insights into the natural history and management of this rare HCM phenotype.

## 2 Materials and methods

This study retrospectively analyzed the clinical data of a patient with HCM. Key diagnostic evaluations were performed as follows:

① Cardiac magnetic resonance imaging (CMR) included the cine sequences and late gadolinium enhancement (LGE) sequences. The diagnostic criterion for left ventricular excessive trabeculation (LVET) was a non-compacted to compacted myocardial thickness ratio >2.3:1 (5).

② Cardiac catheterization was conducted to measure continuous pressure from the left ventricular apex to the aortic root. A left ventricular outflow tract gradient (LVOTG) ≥ 30 mmHg was defined as a clinically significant obstruction.

③ Endomyocardial biopsy (EMB) samples were stained with H&E, Masson trichrome, Congo red, and periodic acid-Schiff (PAS), and independently evaluated by two senior pathologists.

④ Genetic testing was performed using next-generation sequencing with a cardiomyopathy-targeted panel. Sequence variants were named following the guidelines of the Human Genome Variation Society (HGVS), and pathogenicity was interpreted based on American College of Medical Genetics and Genomics (ACMG) standards (6).

## 3 Case presentation

The patient, a 74-year-old male, first presented with dyspnea on exertion in the winter of 2010. Initially, the symptoms were mild and relieved after rest. In October 2013, he experienced frequent dyspnea at rest and was admitted to our hospital for initial evaluation. He had a 20-year history of well-controlled hypertension, with no smoking, alcohol abuse, or family history of heart disease or sudden cardiac death. Laboratory tests, including serum and urine free light chain, immunofixation electrophoresis, and *α*-galactosidase activity, were unremarkable. Transthoracic echocardiography showed diffuse and symmetric left ventricular hypertrophy, with a left ventricular posterior wall thickness (LVPWT) of 23 mm and interventricular septal thickness (IVST) of 24 mm. The left atrial anterior-posterior diameter (LAAPD) was enlarged at 55 mm, while the left ventricular end-diastolic diameter (LVEDD) was 37 mm and left ventricular end-diastolic volume (LVEDV) was 58 mL. The left ventricular ejection fraction (LVEF) was 58% and E/A ratio was 0.70. A resting LVOTG of 36 mmHg was detected, accompanied by systolic anterior motion (SAM) of the mitral anterior leaflet and subvalvular chordae. No regional abnormal ventricular wall motion was observed. Cardiac catheterization demonstrated a resting LVOTG of 45 mmHg. After excluding secondary left ventricular hypertrophy, he was diagnosed with hypertrophic obstructive cardiomyopathy and treated with metoprolol tartrate and diltiazem hydrochloride.

**Figure 1:**
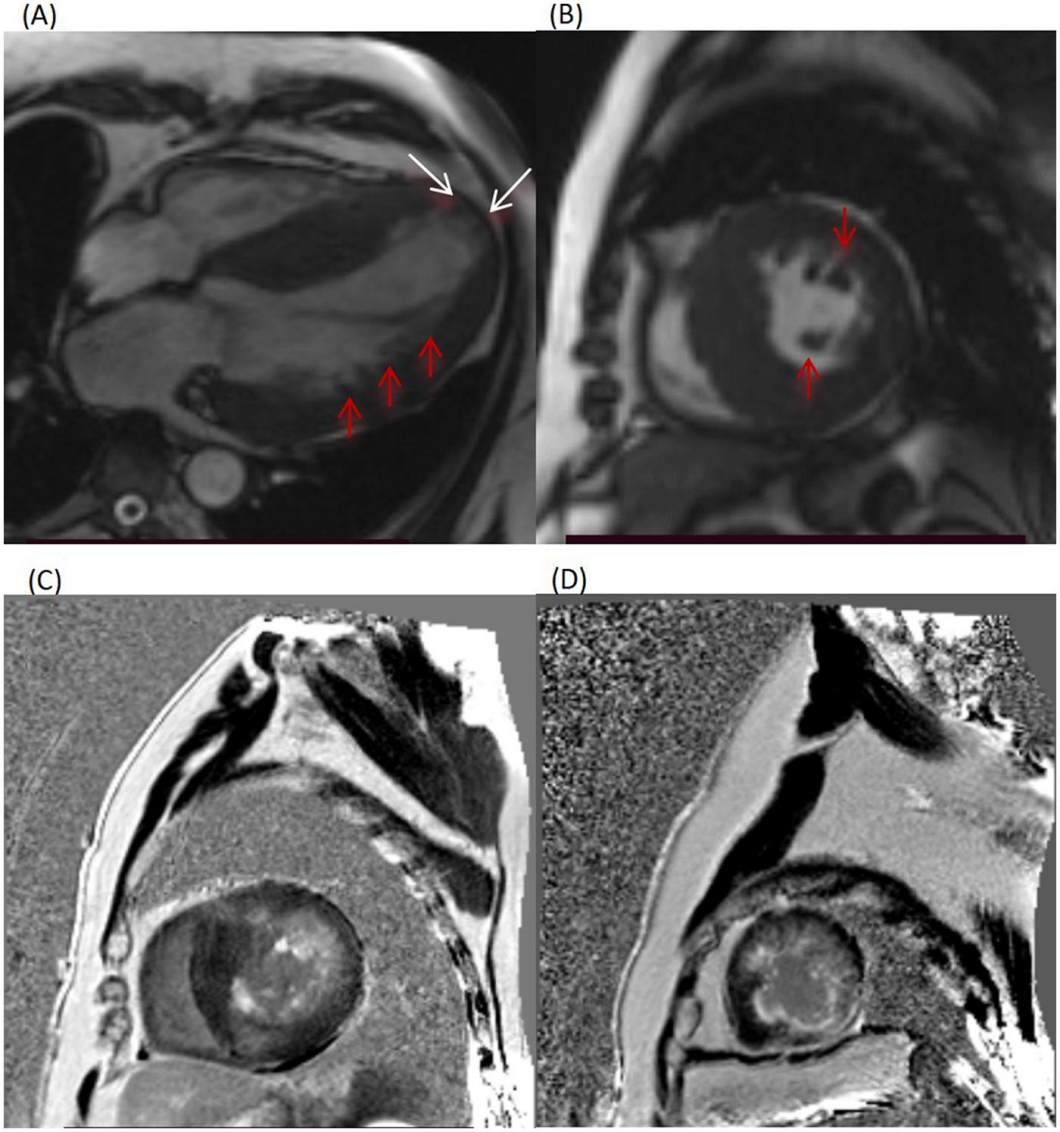
Cardiac magnetic resonance (CMR) images. **(A,B)** Long-axis and short-axis cardiac chamber views revealed symmetric hypertrophy of the left ventricular (LV) free wall and interventricular septum (maximum thickness, 27 mm) and left atrial dilatation (transverse diameter, 69 mm). The LV apex manifested as blunting with thinning, hypokinesis, and focal bulging (white arrows). Marked myocardial trabeculation was noted in the mid-distal LV lateral wall and apical myocardium (red arrows), with a trabeculated-to-compacted myocardial thickness ratio of 2.32:1. **(C,D)** Late gadolinium enhancement (LGE) sequences showed extensive LGE involving the subendocardial and mid-myocardial layers of the interventricular septum, LV free wall, and papillary muscles, indicative of myocardial fibrosis.

In 2015, the patient developed paroxysmal palpitations. A 24 h Holter monitoring (ECG) revealed non-sustained ventricular tachycardia (VT) with R-on-T. CMR revealed left ventricular (12.3 × 4.5 cm) and atrial (6.9 × 5.1 cm) enlargement, diffuse left ventricular hypertrophy (maximal thickness 27 mm), left ventricular outflow tract obstruction, and extensive LGE. The apical myocardium exhibited blunting, thinning, hypokinesis, and mild bulging with excessive trabeculation (Figure 1). After evaluation by the cardiac surgery team, surgical options including the septal myectomy and alcohol septal ablation were recommended (7), but the patient refused surgery due to concerns about intraoperative and postoperative trauma and associated risks. Furthermore, his symptoms were alleviated following the adjustment of pharmacotherapy, which led him to subjectively believe that his condition was well-controlled with medical treatment alone. Given the high sudden cardiac death risk, a rate-responsive double-chamber ICD was implanted. Considering the high risk of thromboembolism, rivaroxaban was added for anticoagulation.

**Figure 2:**
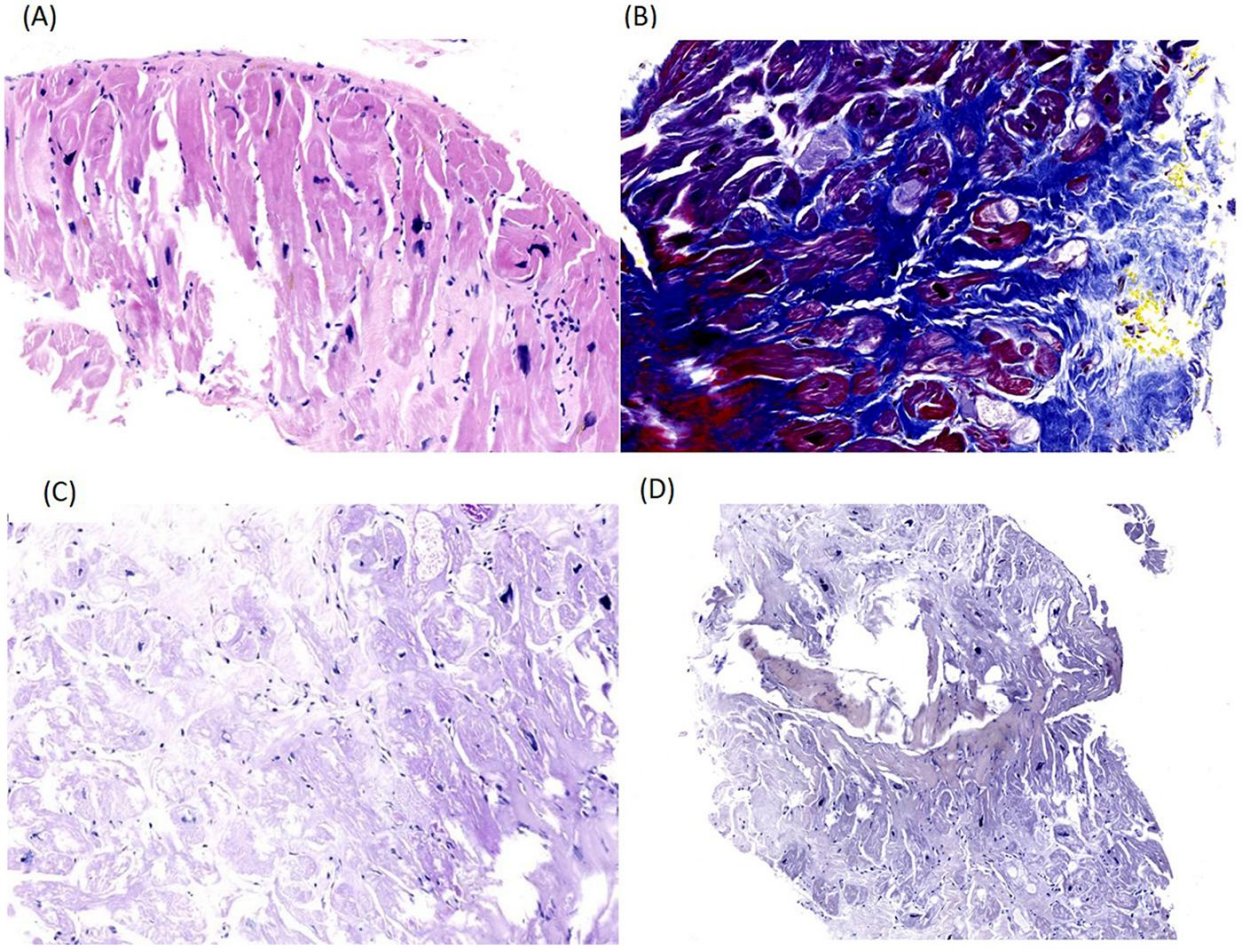
Endomyocardial biopsy of the middle segment of the left ventricular inferior wall. **(A)** Hematoxylin-Eosin (HE) staining: myocardial cell hypertrophy (cell diameter 45–55 μm, normal reference 20–30 μm) and disorganized arrangement; **(B)** Masson staining: interstitial fibrous tissue proliferation; **(C)** Periodic Acid-Schiff (PAS) staining: negative, excluding glycogen storage disease; **(D)** Congo red staining: negative, excluding cardiac amyloidosis.

In 2018, the patient repeatedly experienced paroxysmal palpitations and dyspnea, and ICD programming suggested paroxysmal atrial fibrillation. Repeat cardiac catheterization showed no pressure gradient from LV outflow tract to the aorta. EMB revealed myocardial cell hypertrophy, disorganized arrangement, and interstitial fibrosis, with negative PAS and Congo red staining (Figure 2). Echocardiography showed a negative SAM, focal apical bulging, wall thinning, and apical akinesia. From 2019 to 2024, he was hospitalized three times for acute heart failure decompensation, with progressive decline in LVEF. Heart transplantation was evaluated. However, given the patient's advanced age and comorbidities, the clinical benefits of heart transplantation would be limited (8, 9). In addition, the patient refused heart transplantation due to concerns regarding the highly invasive nature of the transplant surgery and the adverse effects of long-term postoperative immunosuppressive therapy. His regimen was optimized for end-stage heart failure, combined with cardiac rehabilitation.

Furthermore, from August 2021 to the present, the patient experienced seven ICD shocks. Pre-shock symptoms included chest pain, dizziness, and fatigue. All shocks were confirmed as appropriate therapies by programmed device data analysis and comprehensive clinical evaluation. Six episodes of sustained VT were terminated by anti-tachycardia pacing (ATP), and one episode of ventricular fibrillation was treated with a high-energy shock. In 2024, genetic testing of peripheral blood identified a heterozygous missense variant: NM_000256.3: exon30: c.3343G> A: p.Val1115Ile. Regrettably, the patient's first-degree relatives declined genetic testing due to financial constraints.

The patient's most recent follow-up was in June 2025. He could perform basic daily activities but rarely engaged in outdoor activities due to fear of ICD shocks. Intermittent palpitations resolved spontaneously with rest. Transthoracic echocardiography revealed the LVEF of 31%. He received optimized conservative treatment for end-stage heart failure, including metoprolol succinate, sacubitril/valsartan, spironitctone, torsemide, amiodarone, and rivaroxaban, with the goal of preserving quality of life. The clinical course and echocardiographic data are summarized in Table 1, 2, respectively.

**Table 1:** Timeline of clinical course and management.

Time	Clinical Manifestations	Laboratory and imaging examination	Treatment
2010–2015	Paroxysmal dyspnea;	Blood/urine free light chain and immunofixation electrophoresis, blood *α*-galactosidase: No abnormalities detected.	Metoprolol Tartrate and Diltiazem Hydrochloride.
	NYHA functional classⅡ.	2. Cardiac catheterization: LVOTG, 45 mmHg.	
2015–2018	New-onset paroxysmal palpitations;	24 h Holter monitoring: Non-sustained VT with R-on-T.	Declined surgical intervention;
	NYHA functional class Ⅱ∼Ⅲ.	2. CMR (2015): LV and left atrial enlargement; diffuse hypertrophy of LV free wall and interventricular septum (maximum thickness 27 mm); LV outflow tract obstruction (peak velocity under aortic valve 2.24 m/s); LGE in interventricular septum, subendocardial and mid-myocardial layers of LV and papillary muscles; Blunting, thinning, hypokinesis and focal mild bulging of apical myocardium, suggesting early ventricular aneurysm formation; Excessive myocardial trabeculation (trabeculated: compacted myocardial thickness ratio 2.32:1)	2. ICD implantation (2015);
			3. Rivaroxaban was added for anticoagulation.
2018–2021	Paroxysmal palpitation and deteriorating dyspnea.	1. ICD programming: Paroxysmal atrial fibrillation. 2. Cardiac catheterization: No pressure gradient was detected between the left ventricular outflow tract and the aorta.	Metoprolol succinate, Amiodarone hydrochloride, Sacubitril valsartan sodium, Spironolactone, Rivaroxaban, Torsemide.
	NYHA functional class III∼Ⅳ.	3. Myocardial biopsy: Cardiomyocyte hypertrophy, disorganized arrangement, and interstitial fibrous tissue proliferation; PAS staining and Congo red staining were negative.	
2021–2024	Frequent ICD shocks (7 times).	ICD programming: Sustained VT/ventricular fibrillation, with all shocks confirmed as appropriate therapies.	The previous combined medication regimen was continued, with dosages optimized to symptomatically control heart failure and cardiac arrhythmias.
	NYHA functional class III∼IV.	2. Genetic testing: A heterozygous missense variant of the MYBPC3 gene (c.3343G> A:p.Val1115Ile).	

NYHA, New York Heart Association; LVOTG, left ventricular outflow tract gradient; CMR, cardiac magnetic resonance; LV, left ventricular; LGE, late gadolinium enhancement; ICD, implantable cardioverter-defibrillator.

**Table 2:** Transthoracic echocardiography results.

Time	LVPWT (mm)	IVST (mm)	LAAPD (mm)	LVEDD (mm)	LVEDV (mL)	LVEF (%)	E/A	LVOTG (mmHg)	SAM	Abnormal ventricular wall motion
2013.11	23	24	55	37	58	58	0.70	36	Positive	None
2015.12	23	24	50	38	62	56	0.80	36	Positive	None
2018.12	16	22	54	55	147	48	1.22	10	Negative	Focal bulging, wall thinning, and akinesis at the LV apex.
2019.06	16	22	58	49	113	48	1.10	4	Negative	
2021.05	14	17	53	56	154	35	0.80	/	Negative	
2022.07	14	17	53	56	154	34	1.17	/	Negative	
2024.06	13	14	55	60	180	31	1.22	/	Negative	

LVPWT, left ventricular posterior wall thickness; IVST, interventricular septal thickness; LAAPD, left atrial anteroposterior diameter; LVEDD, left ventricular end-diastolic diameter; LVEDV, left ventricular end-diastolic volume; LVEF, left ventricular ejection fraction; E/A, early diastolic filling velocity/late diastolic filling velocity; LVOTG, left ventricular outflow tract gradient; SAM, systolic anterior motion (of the mitral valve).

## 4 Discussion

HCM is a common hereditary cardiomyopathy diagnosed by 2D echocardiography or CMR based on a maximum end-diastolic left ventricular wall thickness ≥15 mm in any segment (10, 11). The patient had a history of hypertension, but with well-controlled blood pressure during long-term follow-up. His clinical symptoms cannot be explained by hypertension. Left ventricular wall hypertrophy caused by hypertension is typically mild (<15 mm) and rarely accompanied by extensive LGE on CMR (12)_._ The patient did not present with the typical clinical manifestations of infiltrative cardiomyopathy. Laboratory tests and EMB findings excluded infiltrative cardiomyopathies (13, 14). In addition, excessive trabeculation on CMR was considered more likely secondary to myocardial fibrosis, wall thinning, and mechanical stress changes associated with HCM and LVAA, rather than an isolated primary left ventricular non-compaction (LVNC) (5, 15). The CMR images showed significant LV hypertrophy involving multiple segments, with the ratio of interventricular septal thickness to LV posterior wall thickness <1.3. This pattern of extensive involvement of multiple ventricular walls is distinctly different from the phenotype of focal or segmental HCM. Thus, the patient was diagnosed with diffuse hypertrophic obstructive cardiomyopathy.

Iacopo Olivotto et al. proposed four clinical stages of HCM: non-hypertrophic (subclinical) HCM, classic HCM, adverse remodeling, and significant dysfunction (16). At presentation, the patient already had classic HCM with adverse cardiac remodeling. Despite optimal medical therapy and ICD implantation, he developed obstruction regression and ventricular wall thinning, progressing to severe cardiac dysfunction. We postulate that such rapid progression may result from multiple factors. First, the patient was diagnosed with diffuse hypertrophic cardiomyopathy. Unlike classic asymmetric septal hypertrophy, diffuse myocardial hypertrophy causes a marked reduction in left ventricular cavity size and severe impairment of myocardial compliance. This not only results in diastolic dysfunction but also disrupts myocardial oxygen supply–demand balance. Combined with coronary wall thickening and luminal narrowing, it markedly increases the risk of myocardial ischemia. Repeated ischemia leads to cardiomyocyte loss and fibrosis, which represent a key pathological substrate for arrhythmias. The presence of LVAA further increased regional stress, exacerbating fibrosis, arrhythmia risk, and electrical instability (4, 17). Additionally, the patient declined surgical intervention in 2015, thus missing the optimal opportunity to relieve early-stage LVOT obstruction and delay adverse myocardial remodeling. Studies have demonstrated that early relief of LVOT obstruction can reduce myocardial ischemia and fibrotic progression, and improve survival outcomes (18). Lastly, the patient was already 63 years old at the time of diagnosis. Advanced age itself impairs myocardial repair capacity and disrupts collagen metabolism homeostasis, thus accelerating the progression of myocardial fibrosis. Meanwhile, the patient had a 20-year history of hypertension. Although blood pressure was well-controlled, previous studies have indicated that hypertension comorbidity is associated with an adverse phenotype in HCM patients, including reduced LVEF and impaired myocardial strain (19).

LVAA is a rare complication and an independent risk factor in HCM. The formation of LVAA will increase the risk of SCD, ventricular arrhythmia, heart failure, and stroke (4). Most LVAA occurs in asymmetric obstructive HCM, often related to midventricular obstruction, abnormal mid-left ventricular blood flow, and reduced subendocardial blood perfusion (20). In this patient, however, LVAA developed despite symmetric hypertrophy and only mild early LVOT obstruction (peak gradient 36 mmHg), and persisted after obstruction resolved. Therefore, outflow tract obstruction cannot wholly explain the formation of LVAA in this case. It is also considered to be related to factors such as severe myocardial fibrosis, myocardial injury and reperfusion injury, hemodynamic changes, and increased cardiac load. This patient suffered from atrial fibrillation. Furthermore, Patients with LVAA are at an increased risk of intraventricular thrombus formation. Therefore, anticoagulation therapy is essential for this patient.

About 30%–60% of patients with HCM have identifiable pathogenic or possible pathogenic genetic variants, most of which occur in myosin-binding protein C (MYBPC3) and myosin heavy chain (MYH7). The latest meta-analysis showed that MYBPC3 penetrance was about 55% in HCM patients, and the average age of onset was 41 years (21). According to the ACMG guidelines, the MYBPC3 c.3343G> A variant has been classified as a Variant of Uncertain Significance (VUS). In the gnomAD database, its allele frequency in the general population is approximately 0.0013% (6). The detection of this variant in the patient might show a certain correlation with the clinical phenotype. Compared with HCM patients carrying pathogenic/likely pathogenic gene variants, those with variants of VUS have a later age of onset, with a more prominent increase in left ventricular wall thickness but a lower interventricular septal thickness. This may explain the clinical phenotype of diffuse left ventricular hypertrophy in this patient (22). About 90% of the mutations in the MYBPC3 gene are truncating, while this patient carries a less common heterozygous missense mutation. Recent studies have suggested that specific missense mutations may also be associated with a higher clinical risk in HCM patients. Certain MYBPC3 missense mutations located in key functional domains may cause subdomain misfolding, interfere with the assembly and function of sarcomeric proteins, and thereby potentially lead to myocardial structural disarray and electrical instability (23, 24). However, this remains speculative for the specific variant in this case. Therefore, the correlation between this VUS and the patient's severe clinical manifestations requires further validation through larger cohort studies and functional assays.

The CMR images of this patient indicated the presence of LVET. A relevant expert consensus in 2023 proposed replacing the term “LVNC” with “excessive trabeculation”. Specifically, the extent of trabeculated vs. compact myocardium in adults is determined by the differential growth of each myocardial layer (5). Excessive trabeculation is a ventricular phenotype identified by imaging of echocardiography or CMR. In this case, CMR showed that the ratio of non-compacted to compacted myocardial thickness was 2.32:1. Furthermore, this abnormal finding was closely associated with HCM-related myocardial remodeling rather than representing an isolated primary lesion. Therefore, the term “LVET” is more consistent with the patient's pathophysiological characteristics. The patient exhibited myocardial thinning and abnormally prominent trabeculations in the mid-distal left ventricular lateral wall and apex. Excessive myocardial trabeculation in this region is significantly associated with an increased risk of heart failure and ventricular arrhythmias (15). CMR is helpful for the early detection of abnormal findings such as excessive myocardial trabeculation and apical ventricular aneurysm, thereby enabling early clinical intervention (5, 25).

## 5 Patient perspective and implications

The long-term management of this case reflects both the disease trajectory and the patient's personal experiences and quality of life challenges with a chronic progressive cardiomyopathy. ICD-related anxiety was prominent. Frequent appropriate ICD shocks effectively terminated life-threatening arrhythmias but caused persistent fear, leading the patient to limit outdoor activities and social involvement. Studies have shown that ICD shocks are significantly associated with anxiety, depression, and reduced quality of life (14). Regular psychosocial assessment and optimized ICD programming are recommended to alleviate anxiety and improve well-being. In addition, the patient declined surgical septal reduction and heart transplantation due to concerns about invasiveness and long-term risks. These decisions reflected the importance of shared decision-making and value-based communication. Clinicians should balance guideline-directed recommendations with patient priorities.

Finally, this case also offers additional implications for clinical practice: for patients with diffuse hypertrophic cardiomyopathy, routine CMR, genetic testing, and close follow-up are recommended for early complication detection. End-stage management requires a multidisciplinary approach, including optimized medical therapy, ICD programming, and heart transplantation evaluation when appropriate.

## 6 Limitations

This study has several limitations. First, the MYBPC3 c.3343G> A (p. Val1115Ile) variant is currently classified as a VUS. Although this variant is consistent with part of the patient's clinical phenotype, its pathogenicity remains to be confirmed by family cosegregation analysis and functional studies. Second, given the long-term follow-up of more than a decade, only paper-based echocardiographic reports were available for the initial obstructive hypertrophy stage, and original digital imaging data were no longer accessible. This limited retrospective quantitative analysis and may have hindered complete characterization of disease progression. Third, early CMR in 2015 demonstrated atypical features of an apical aneurysm, including apical hypokinesis, regional thinning, mild bulging, and corresponding LGE. However, it lacked the typical saccular bulge and paradoxical wall motion of classic apical aneurysms. Although echocardiography later confirmed the apical aneurysm, these atypical early CMR findings may have affected initial phenotypic assessment. Subsequent cardiac function deterioration made further CMR impossible, limiting serial monitoring of aneurysm progression. Finally, the patient's refusal of surgery prevented the assessment of the efficacy of surgical treatment in this case.

## Data Availability

The original contributions presented in the study are included in the article; further inquiries can be directed to the corresponding author/s.

## References

[1] Maron MS Maron BJ Harrigan C Buros J Gibson CM Olivotto I Hypertrophic cardiomyopathy phenotype revisited after 50 years with cardiovascular magnetic resonance. J Am Coll Cardiol. (2009) 54(3):220–8. 10.1016/j.jacc.2009.05.006 19589434

[2] Delcrè SD Di Donna P Leuzzi S Miceli S Bisi M Scaglione M Relationship of ECG findings to phenotypic expression in patients with hypertrophic cardiomyopathy: a cardiac magnetic resonance study. Int J Cardiol. (2013) 167(3):1038–45. 10.1016/j.ijcard.2012.03.074 22464482

[3] Muresan ID Agoston-Coldea L. Phenotypes of hypertrophic cardiomyopathy: genetics, clinics, and modular imaging. Heart Fail Rev. (2021) 26(5):1023–36. 10.1007/s10741-020-09931-1 32040801

[4] Lee DZJ Montazeri M Bataiosu R Hoss S Adler A Nguyen ET Clinical characteristics and prognostic importance of left ventricular apical aneurysms in hypertrophic cardiomyopathy. JACC Cardiovasc Imaging. (2022) 15(10):1696–711. 10.1016/j.jcmg.2022.03.029 36202449

[5] Petersen SE Jensen B Aung N Friedrich MG McMahon CJ Mohiddin SA Excessive trabeculation of the left ventricle: JACC: cardiovascular imaging expert panel paper. JACC Cardiovasc Imaging. (2023) 16(3):408–25. 10.1016/j.jcmg.2022.12.026 36764891 PMC9988693

[6] Richards S Aziz N Bale S Bick D Das S Gastier-Foster J Standards and guidelines for the interpretation of sequence variants: a joint consensus recommendation of the American college of medical genetics and genomics and the association for molecular pathology. Genet Med. (2015) 17(5):405–24. 10.1038/gim.2015.30 25741868 PMC4544753

[7] Elliott PM Anastasakis A Borger MA Borggrefe M Cecchi F Charron P 2014 ESC guidelines on diagnosis and management of hypertrophic cardiomyopathy: the task force for the diagnosis and management of hypertrophic cardiomyopathy of the European Society of Cardiology (ESC). Eur Heart J. (2014) 35(39):2733–79. 10.1093/eurheartj/ehu284 25173338

[8] Seferović PM Polovina M Bauersachs J Arad M Ben Gal T Lund LH Heart failure in cardiomyopathies: a position paper from the heart failure association of the European Society of Cardiology. Eur J Heart Fail. (2019) 21(5):553–76. 10.1002/ejhf.1461 30989768

[9] Velleca A Shullo MA Dhital K Azeka E Colvin M DePasquale E The international society for heart and lung transplantation (ISHLT) guidelines for the care of heart transplant recipients. J Heart Lung Transplant. (2023) 42(5):e1–e141. 10.1016/j.healun.2022.10.015 37080658

[10] Maron BJ Desai MY Nishimura RA Spirito P Rakowski H Towbin JA Diagnosis and evaluation of hypertrophic cardiomyopathy: JACC state-of-the-art review. J Am Coll Cardiol. (2022) 79(4):372–89. 10.1016/j.jacc.2021.12.002 35086660

[11] Ommen SR Ho CY Asif IM Balaji S Burke MA Day SM 2024 AHA/ACC/AMSSM/HRS/PACES/SCMR guideline for the management of hypertrophic cardiomyopathy: a report of the American Heart Association/American College of Cardiology joint committee on clinical practice guidelines. Circulation. (2024) 149(23):e1239–e311. 10.1161/CIR.0000000000001250 38718139

[12] Han Y Li Y Wu Z Pei Y Lu S Yu H Progress in diagnosis and treatment of hypertension combined with left ventricular hypertrophy. Ann Med. (2024) 56(1):2405080. 10.1080/07853890.2024.2405080 39301864 PMC11418038

[13] Rubin J Maurer MS. Cardiac amyloidosis: overlooked, underappreciated, and treatable. Annu Rev Med. (2020) 71:203–19. 10.1146/annurev-med-052918-020140 31986086

[14] van der Lingen ACJ Rijnierse MT Hooghiemstra AM Elshout S van Halm VP Batelaan NM The link between cardiac status and depression and anxiety in implantable cardioverter defibrillator patients: design and first results of the PSYCHE-ICD study. J Psychosom Res. (2023) 167:111182. 10.1016/j.jpsychores.2023.111182 36801661

[15] Zhang Y Zhou D Zhu Y Huang Q Wang J Zhang C Patterns of left ventricular trabeculation in hypertrophic cardiomyopathy. BMC Med. (2025) 23(1):318. 10.1186/s12916-025-04142-7 40442667 PMC12123774

[16] Olivotto I Cecchi F Poggesi C Yacoub MH. Patterns of disease progression in hypertrophic cardiomyopathy: an individualized approach to clinical staging. Circ Heart Fail. (2012) 5(4):535–46. 10.1161/CIRCHEARTFAILURE.112.967026 22811549

[17] Papanastasiou CA Zegkos T Karamitsos TD Rowin EJ Maron MS Parcharidou D Prognostic role of left ventricular apical aneurysm in hypertrophic cardiomyopathy: a systematic review and meta-analysis. Int J Cardiol. (2021) 332:127–32. 10.1016/j.ijcard.2021.03.056 33794232

[18] Schaff HV Wei X. Contemporary surgical management of hypertrophic cardiomyopathy. Ann Thorac Surg. (2024) 117(2):271–81. 10.1016/j.athoracsur.2023.10.026 37914148

[19] Yang Z Zhang TY Gui FD Yao FY Long YT Wen M Hypertension and its association to phenotype on left ventricular function in hypertrophic cardiomyopathy patients assessed by cardiovascular magnetic resonance imaging. Clin Radiol. (2024) 79(12):941–9. 10.1016/j.crad.2024.08.028 39304482

[20] Strachinaru M Huurman R Bowen DJ Schinkel AFL Hirsch A Michels M. Relation between early diastolic mid-ventricular flow and elastic forces indicating aneurysm formation in hypertrophic cardiomyopathy. J Am Soc Echocardiogr. (2022) 35(8):846–56.e2. 10.1016/j.echo.2022.04.010 35489541

[21] Topriceanu CC Pereira AC Moon JC Captur G Ho CY. Meta-Analysis of penetrance and systematic review on transition to disease in genetic hypertrophic cardiomyopathy. Circulation. (2024) 149(2):107–23. 10.1161/CIRCULATIONAHA.123.065987 37929589 PMC10775968

[22] Curran L de Marvao A Inglese P McGurk KA Schiratti P-R Clement A Genotype-Phenotype taxonomy of hypertrophic cardiomyopathy. Circ Genom Precis Med. (2023) 16(6):e004200. 10.1161/CIRCGEN.123.004200 38014537 PMC10729901

[23] Thompson AD Helms AS Kannan A Yob J Lakdawala NK Wittekind SG Computational prediction of protein subdomain stability in MYBPC3 enables clinical risk stratification in hypertrophic cardiomyopathy and enhances variant interpretation. Genet Med. (2021) 23(7):1281–7. 10.1038/s41436-021-01134-9 33782553 PMC8257482

[24] Helms AS Thompson AD Glazier AA Hafeez N Kabani S Rodriguez J Spatial and functional distribution of MYBPC3 pathogenic variants and clinical outcomes in patients with hypertrophic cardiomyopathy. Circ Genom Precis Med. (2020) 13(5):396–405. 10.1161/CIRCGEN.120.002929 32841044 PMC7676622

[25] Maron MS Finley JJ Bos JM Hauser TH Manning WJ Haas TS Prevalence, clinical significance, and natural history of left ventricular apical aneurysms in hypertrophic cardiomyopathy. Circulation. (2008) 118(15):1541–9. 10.1161/CIRCULATIONAHA.108.781401 18809796

